# Knowledge, attitude, and practice toward non-nutritive sweeteners among the population with reduced sugar intake requirement

**DOI:** 10.3389/fnut.2023.1268599

**Published:** 2024-01-05

**Authors:** Qiao Chen, Yan Zhang, Hui Li

**Affiliations:** ^1^Department of Nutrition, Third Medical Center of PLA General Hospital, Beijing, China; ^2^Department of Clinical Nutrition, Leling People's Hospital, Leling, China

**Keywords:** KAP, non-nutritive sweetener, obesity, overweight, diabetes

## Abstract

**Introduction:**

This study aimed to explore the knowledge, attitude, and practice (KAP) toward non-nutritive sweeteners among a population with reduced sugar intake requirements.

**Methods:**

This cross-sectional study used self-developed questionnaires to collect demographic characteristics and KAP towards non-nutritive sweeteners among respondents with reduced sugar intake requirements, i.e., overweight or obese individuals and patients with pre-diabetes or diabetes.

**Results:**

A total of 639 valid questionnaires were collected, and 51.64% of participants were male. The KAP scores were 7.63 ± 3.58 (range: 0–11), 34.28 ± 7.47 (range: 12–60), and 15.48 ± 3.97 (range: 7–35), respectively. Pearson’s correlation analysis showed that knowledge score was positively correlated with attitude (*r* = 0.229, *p* < 0.001) and practice score (*r* = 0.467, *p* < 0.001), while attitude was positively correlated with practice (*r* = 0.312, *p* < 0.001). The structural equation model showed that knowledge was directly and positively associated with attitude (path coefficient = 0.48, *p* < 0.001) and practice (path coefficient = 0.46, *p* < 0.001). In addition, the attitude was directly and positively associated with practice (path coefficient = 0.12, *p* < 0.001). Besides, diabetes was associated with lower knowledge (path coefficient = −0.81, *p* = 0.038) and practice (path coefficient = −0.42, *p* < 0.041).

**Discussion:**

Population with the reduced sugar intake requirement showed poor knowledge, negative attitudes, and suboptimal practices toward non-nutritive sweeteners. To optimize the utilization of non-nutritive sweeteners in accordance with medical prescriptions, especially for individuals with diabetes, tailored educational interventions may be designed for participants with lower KAP.

## Introduction

Overweight and obesity have become significant global health concerns, constituting risk factors for numerous chronic diseases, including type 2 diabetes, and thus imposing a substantial burden on healthcare systems (1). In China, the prevalence of overweight and obesity among adults has become disturbingly high, with rates of 28.1 and 5.2%, respectively (2). Besides, diabetes mellitus ranks among the top contributors to mortality and disability worldwide. In northeast China, the estimated prevalence of diabetes and pre-diabetes among adults is 9.1 and 19.8%, respectively (3). Therefore, overweight and obese individuals, as well as those diagnosed with pre-diabetes and diabetes, are confronted with significant challenges in weight and blood glucose management, all of which include sugar reduction (4, 5). However, reducing sugar intake while maintaining a satisfying eating experience might pose certain difficulties.

Non-nutritive sweeteners, commonly known as artificial or low-calorie sweeteners, have garnered interest as sugar substitutes due to their low caloric content (6). These sweeteners offer a sweet taste without adding to energy intake, and they have been widely used in food and beverage items. Using non-nutritive sweeteners has been linked to potential advantages, such as weight management and glycemic control (7, 8). Additionally, replacing sugar-sweetened beverages with non-nutritive sweetened alternatives could reduce the risk of diabetes associated with sugar-sweetened beverage consumption by approximately half (9). Nonetheless, a recent World Health Organization (WHO) guideline has challenged the above assumption. Limited evidence supports the long-term reduction in body fat using non-nutritive sweeteners, while an increased risk of type 2 diabetes, cardiovascular diseases, and mortality have been associated with sweeteners in adults (10, 11). Therefore, using non-nutritive sweeteners among those with reduced sugar intake requirements should be taken cautiously.

KAP study toward non-nutritive sweeteners can provide valuable insights into weight management and glycemic control strategies. To date, relevant KAP studies are lacking. Findings from the United Kingdom revealed that approximately half of the participants held a high-risk perception of non-nutritive sweeteners and lacked knowledge of regulations associated with these sweeteners (12). Similarly, research from Iran indicated that around half of the patients with diabetes had moderate knowledge and attitudes toward non-nutritive sweetener consumption (13). Of note, there is a dearth of studies examining these aspects in China. Accordingly, the present study investigated the KAP toward non-nutritive sweeteners among overweight and obese individuals, as well as patients with pre-diabetes and diabetes.

## Materials and methods

### Study design and participants

The cross-sectional survey was conducted among a population with reduced sugar intake requirements between November 2022 and April 2023. In this study, the population with reduced sugar intake requirement was defined as those overweight or obese and patients with pre-diabetes or diabetes. The inclusion criteria were the following: (1) those who met the diagnostic criteria for diabetes mellitus or pre-diabetes, or overweight or obesity; (2) age **≥** 18 years. The exclusion criteria referred to individuals who could not respond and those who provided duplicate or incomplete responses to the questionnaire. The study received ethical approval from the Medical Ethics Committee of the PLA General Hospital (KY2022-025), and informed consent was obtained from all participants.

Diabetes or pre-diabetes was diagnosed following the criteria outlined in the 2022 Chinese Guidelines for the Prevention and Treatment of Hyperglycemia. Patients who met at least one of the following criteria were considered as having diabetes: (1) Diabetic symptom and random blood glucose ≥11.1 mmol/L; (2) Fasting blood glucose ≥7.0 mmol/L; (3) 2-h postprandial blood glucose ≥11.1 mmol/L; (4) random blood glucose ≥11.1 mmol/L for two consecutive days.

Patients who met at least one of the following criteria were considered as having pre-diabetes: (1) fasting blood glucose levels ranging from 6.1–6.9 mmol/L, and 2-h postprandial blood glucose levels below 7.8 mmol/L; or (2) fasting blood glucose levels <6.1 mmol/L, and 2-h postprandial blood glucose ranging from 7.8–11.0 mmol/L (or HbA_1C_ ranging from5.7–6.4%). Participants with a body mass index (BMI) between 24.0–27.9 were classified as overweight, while those with a BMI > 28.0 were categorized as obese (14).

### Questionnaire

The questionnaire was developed based on relevant literature (12, 15). A pilot study was conducted among 82 participants, yielding a Cronbach’s α value of 0.840, which indicated good internal consistency.

The final questionnaire comprised four dimensions, i.e., demographic characteristics (age, gender, weight, height, residence, ethnicity, education, profession, monthly income, diabetes, and pre-diabetes) and KAP. The other in the “Occupation” category included students, those not employed, and retirees. A threshold of 5,000 CNY, which was used in the “Monthly Income (Yuan)” category, represents the lowest income subject to taxation in China. The BMI threshold was based on the Chinese BMI classification where underweight (<18.5 kg/m^2^), normal weight (18.5 to <24 kg/m^2^), overweight (24 to <28 kg/m^2^), and obesity (≥28 kg/m^2^) (16). The duration of diabetes was defined as the period from the first diagnosis to the completion of the questionnaire. The question was structured in a closed-end format to reduce misunderstandings of the participants’ replies. The 5-year intervals were selected based on past literature (17). The knowledge dimension consisted of 10 questions with 17 items, with correct answers awarded 1 point and incorrect or unclear responses scoring 0 points. In the sensitivity analysis, question 7 in the knowledge dimension was removed. The attitude dimension included 12 questions evaluated on a five-point Likert scale, ranging from strongly agree (5 points) to strongly disagree (1 point). Reverse scoring was applied to Questions 3–4. The practice dimension consisted of 10 questions evaluated on a five-point Likert scale. Questions 1–5 were scored in a forward manner, while the remaining questions were analyzed descriptively.

The study sample size was determined according to a previous study (18).


$$n=\frac{z^{2}pq}{e^{2}}$$

where n represents the number of participants, z is 1.96 for a 95% confidence interval, p is the expected proportion, q is 1-p, and e is the margin of error set at 5%. Adopting the conservative approach, 50% was selected as the expected proportion to maximize the sample size. Consequently, the estimated sample size for this study was 384.

The questionnaire was distributed using both online and offline methods. In the offline approach, patients were recruited from the Third Medical Centre of the PLA General Hospital in Beijing, where they were given the questionnaire during inpatient consultations by scan QR code which were posted within nutrition, endocrinology, and weight loss clinics. To address challenges encountered by individuals filling out the questionnaires, proficient research team members conducted alternative face-to-face interviews. For online distribution, the questionnaire link was shared through the researcher’s social media accounts, the diabetes-related WeChat group, and the Clinical Nutrition Network Official Account. Participants were required to provide demographic information, height, weight, and details about their diabetes condition. Participants’ total scores were categorized using predefined cutoffs, where scores <60% were classified as poor/negative/suboptimal, 60–80% as moderate, and > 80% as good/positive/proactive (19).

### Statistical analysis

Stata 17.0 (Stata Corporation, College Station, TX, United States) was utilized for statistical analysis. The continuous data were expressed as mean ± standard deviations (SD) and compared by Student’s *t*-test or one-way ANOVA. Moreover, the categorical data were expressed as n (%). Pearson correlation analysis was conducted to assess the relationship between the KAP dimensions. Path analysis was conducted to examine the interrelationships between KAP scores and the associations between sociodemographic factors and KAP scores. Path analysis was chosen for its capability to test the linear relationships between variables and quantify the direct and indirect effects of variables on each other. Path analysis was employed to test the following hypotheses: (1) frequency of sweets consumption, overweight status, and diabetes and pre-diabetes diagnoses would impact knowledge; (2) knowledge would impact attitude; (3) knowledge would impact practice; (4) attitude would impact practice. In the sensitivity analysis, we removed question 7 in the knowledge dimension. Pearson’s correlation analysis was conducted to assess the robustness of KAP correlations. Moreover, pathway analysis was carried out to explore the influential factors of KAP scores and evaluate KAP relationships. A two-sided *p* < 0.05 was considered statistically significant.

## Results

### Demographic characteristics

A total of 639 questionnaires were collected, of which 457 were valid for formal analysis. Participants were mainly men (51.64%), aged 46.07 ± 13.91 years old. Among the participants, 45.51% were overweight, 37.42% were obese, 44.86% were diagnosed with diabetes, and 8.32% were diagnosed with pre-diabetes. Notably, a daily consumption of sweets was reported by 22.32% of participants (Table 1).

**Table 1:** Participants’ demographic information and KAP scores.

Variables	N (%)	Knowledge score		Attitude score		Practice score	
		Mean ± SD	*P*	Mean ± SD	*P*	Mean ± SD	*P*
Total	457	7.63 ± 3.58		34.28 ± 7.47		15.48 ± 3.97	
Age (years)	46.07 ± 13.91		<0.001		0.394		0.100
<45	221 (48.36)	8.48 ± 3.17		34.58 ± 7.93		15.80 ± 3.89	
≥45	236 (51.64)	6.83 ± 3.76		33.99 ± 7.01		15.19 ± 4.02	
BMI (kg m^−2^)			0.989		0.345		0.179
<18.5	3 (0.66)	8.33 ± 4.16		30.00 ± 5.00		16.67 ± 3.51	
(18.5, 24)	75 (16.41)	7.63 ± 3.57		33.21 ± 7.92		16.37 ± 3.78	
(24, 28)	208 (45.51)	7.61 ± 3.68		34.75 ± 7.27		15.28 ± 3.91	
≥28	171 (37.42)	7.64 ± 3.48		34.25 ± 7.52		15.32 ± 4.09	
Gender			0.859		0.821		0.858
Male	236 (51.64)	7.66 ± 3.61		34.20 ± 7.24		15.45 ± 4.14	
Female	221 (48.36)	7.60 ± 3.56		34.36 ± 7.72		15.52 ± 3.79	
Residence			<0.001		0.053		<0.001
Urban	368 (80.53)	7.99 ± 3.50		34.61 ± 7.68		15.84 ± 3.85	
Non-urban	89 (19.47)	6.12 ± 3.55		32.90 ± 6.35		13.99 ± 4.11	
Ethnicity			0.804		0.614		0.658
Han ethnicity	444 (97.16)	7.64 ± 3.59		34.25 ± 7.49		15.50 ± 3.95	
Minority	13 (2.84)	7.38 ± 3.33		35.31 ± 6.70		15.00 ± 4.58	
Education			<0.001		0.031		<0.001
Primary school and below	16 (3.50)	4.00 ± 3.25		30.56 ± 6.02		12.13 ± 4.80	
Middle school/high school/technical secondary school	109 (23.85)	6.09 ± 3.81		33.34 ± 6.86		13.96 ± 4.27	
Junior college/undergraduate	301 (65.86)	8.18 ± 3.26		34.94 ± 7.67		16.18 ± 3.53	
Postgraduate and above	31 (6.78)	9.58 ± 2.90		33.06 ± 7.46		15.74 ± 4.24	
Occupation			<0.001		0.439		<0.001
Regular employees	269 (58.86)	8.52 ± 3.12		34.65 ± 7.32		16.31 ± 3.51	
Part-time employees OR freelance	63 (13.79)	6.16 ± 3.95		33.68 ± 7.68		13.27 ± 4.68	
Other	125 (27.35)	6.46 ± 3.75		33.77 ± 7.68		14.82 ± 3.98	
Monthly income (Yuan)			<0.001		0.196		<0.001
<5,000	141 (30.85)	6.13 ± 3.67		33.38 ± 8.00		14.09 ± 4.29	
5,000–10,000	147 (32.17)	7.66 ± 3.61		34.42 ± 6.88		15.61 ± 3.73	
10,000–20,000	106 (23.19)	8.91 ± 3.00		35.42 ± 6.89		16.80 ± 3.70	
>20,000	63 (13.79)	8.75 ± 2.97		34.02 ± 8.32		16.08 ± 3.23	
Social security			0.761		0.689		0.722
Yes	454 (99.34)	7.63 ± 3.58		34.26 ± 7.48		15.49 ± 3.98	
No	3 (0.66)	7.00 ± 4.36		36.00 ± 6.24		14.67 ± 1.53	
Commercial medical insurance			0.135		0.954		0.027
Yes	78 (17.07)	8.18 ± 3.47		34.23 ± 6.67		16.38 ± 3.96	
No	379 (82.93)	7.51 ± 3.60		34.28 ± 7.63		15.30 ± 3.95	
Diabetes			0.028		0.271		0.074
Yes	205 (44.86)	7.22 ± 3.62		33.85 ± 7.51		15.85 ± 4.17	
No	252 (55.14)	7.96 ± 3.52		34.62 ± 7.43		15.18 ± 3.77	
Duration of diabetes (years)			0.104		0.395		0.194
0–5	108 (52.68)	7.80 ± 3.25		34.51 ± 7.53		16.36 ± 3.74	
6–10	48 (23.41)	6.79 ± 4.12		33.50 ± 7.98		15.46 ± 4.98	
11–15	27 (13.17)	6.30 ± 3.98		31.78 ± 7.38		15.67 ± 4.52	
16 and above	22 (10.73)	6.45 ± 3.53		33.91 ± 6.35		14.41 ± 3.65	
Pre-diabetes			0.855		0.760		0.874
Yes	38 (8.32)	7.53 ± 4.08		33.92 ± 6.97		15.58 ± 3.61	
No	419 (91.68)	7.64 ± 3.54		34.31 ± 7.52		15.47 ± 4.00	
Taking oral hypoglycemic drugs			0.055		0.328		0.054
Yes	174 (38.07)	7.22 ± 3.54		33.84 ± 7.71		15.94 ± 3.97	
No	283 (61.93)	7.88 ± 3.59		34.54 ± 7.31		15.20 ± 3.95	
Injecting insulin			0.246		0.917		0.773
Yes	56 (12.25)	7.11 ± 3.87		34.18 ± 7.95		15.63 ± 4.30	
No	401 (87.75)	7.70 ± 3.54		34.29 ± 7.41		15.46 ± 3.92	
Frequency of sweets consumption			0.034		0.029		0.340
More than once a day	18 (3.94)	6.17 ± 3.94		32.39 ± 6.75		14.06 ± 4.32	
Almost every day	102 (22.32)	8.38 ± 3.48		35.52 ± 7.46		15.85 ± 3.96	
Every 1–2 days	82 (17.94)	7.95 ± 3.24		35.74 ± 7.11		15.78 ± 3.62	
Occasionally	189 (41.36)	7.40 ± 3.59		33.27 ± 7.16		15.23 ± 4.00	
Hardly ever	66 (14.44)	7.11 ± 3.84		33.92 ± 8.51		15.64 ± 4.18	

### Knowledge

The participants achieved an average knowledge score of 7.63 ± 3.58 (range: 0–11) (Table 1). Knowledge scores significantly differed among participants from different age groups, residence, education, employment, diabetes, monthly income, and frequency of sweets consumption (*p* < 0.001). Specifically, only 15.54% of participants correctly identified the natural sweeteners commonly used in China (K3). Around one-third of participants were aware of the potential benefits of using non-nutritive sweeteners, including weight control (33.04%), reduced tooth decay risk (37.86%), reduced chronic diseases risk (34.57%), and stable blood sugar (35.45%), (K7). Conversely, the majority (78.34%) recognized that non-nutritive sweeteners are commonly found in foods like chewing gum, cakes, and beverages (K8) (Supplementary Table S1).

### Attitude

The participants scored 34.28 ± 7.47 (range: 12–60) on attitude (Table 1). Attitude scores significantly differed among participants with different education and frequency of sweets consumption (both *p* < 0.05). Notably, a considerable proportion of participants (66.52%) expressed concerns about the potential health impact of consuming non-nutritive sweeteners (A4). Conversely, only 12.69% of participants agreed that sweeteners were completely safe for consumer health (A10). Additionally, merely 21.66% of individuals agreed that non-nutritive sweeteners could fully replace sucrose in food products (A12) (Supplementary Table S2).

### Practice

**Figure 1:**
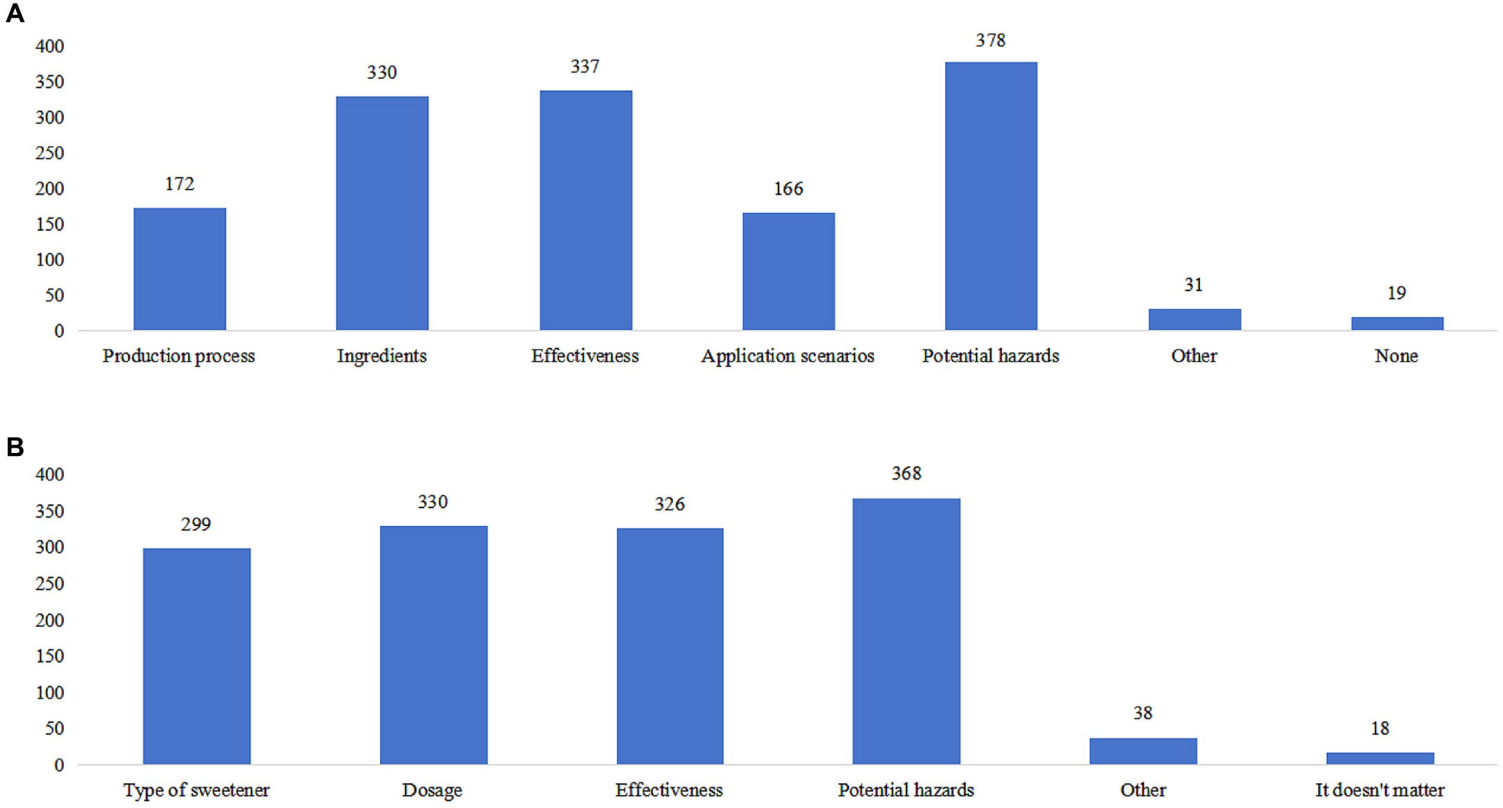
The distribution of information on sweeteners for which participants demonstrated interest in acquiring knowledge. **(A)** information of sweeteners the participants would like to learn more about. **(B)** information of sweeteners the participants would like to find on the packaging of a sweetener product.

**Figure 2:**
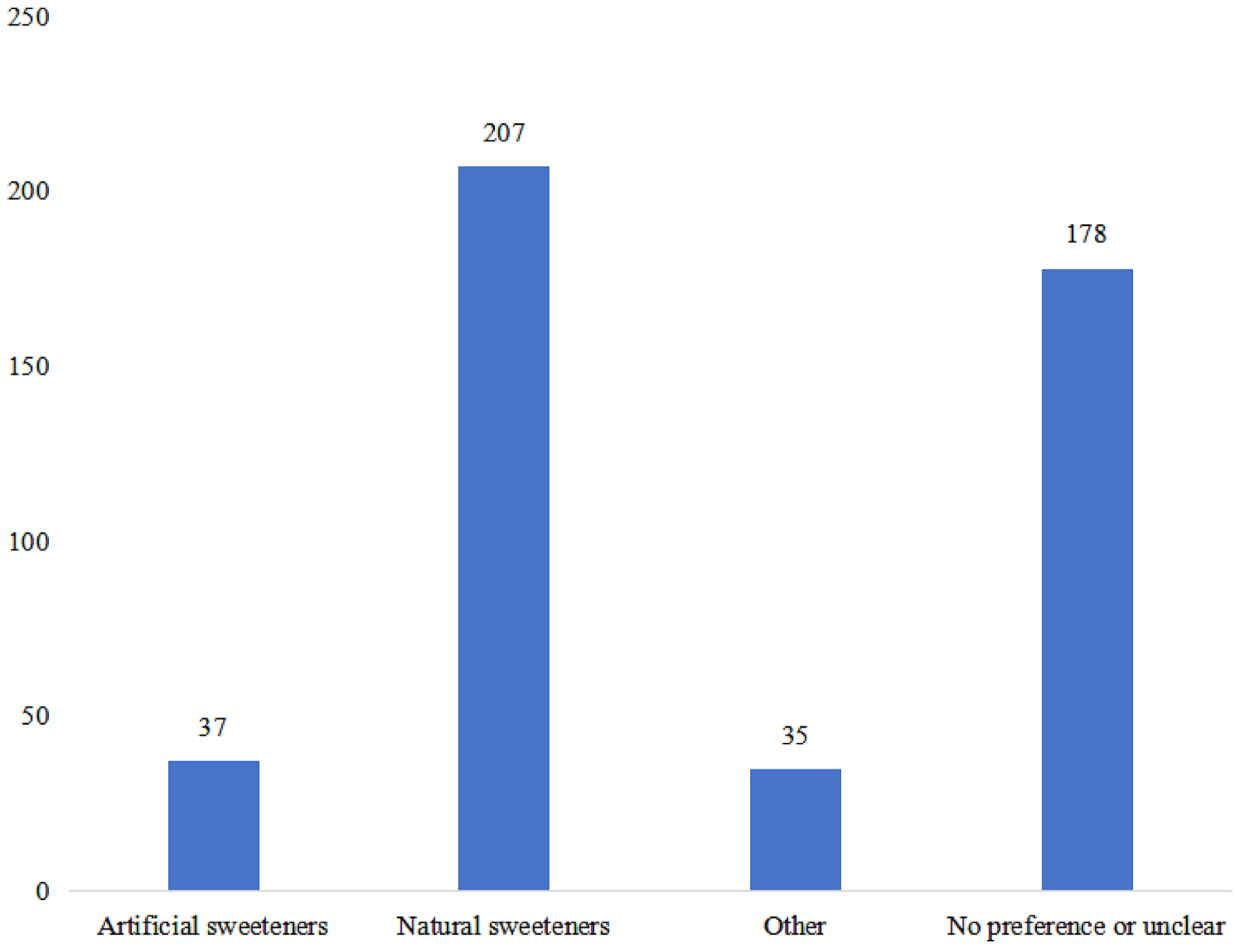
The selection of sweeteners by the participants.

The practice score yielded a value of 15.48 ± 3.97 (range: 7–35), with significant differences among participants with different residence, education, occupation, monthly income, and commercial medical insurance (all *p* < 0.05) (Table 1). Different proportion of participants (ranging from 17.51 to 65.64%) reported “Always” or “Often” in recommended practices. The majority (65.64%) indicated a preference for foods labeled as “zero sugar” or “zero energy” (P5). Furthermore, 52.3% expressed more significant concern about the functional properties of non-nutritive sweeteners when choosing sweetened foods (P7). However, only 17.51% reported frequent consumption of non-nutritive sweeteners as a substitute for sucrose in their diet (P1) (Supplementary Table S3). The highest percentage of participants’ interest in learning about non-nutritive sweeteners was focused on potential hazards (82.71%), followed by effectiveness (73.74%) and ingredients (72.21%) (Figure 1). Additionally, a significant proportion of participants expressed interest in knowing the potential hazards (80.53%), dosage (72.21%), effectiveness (71.33%), and type of non-nutritive sweeteners (65.43%) displayed on sweetener product packaging (Figure 1). Furthermore, natural sweeteners (45.30%), such as stevia and mogroside, were the most preferred sweetener products (Figure 2).

### Correlations and path analysis

**Figure 3:**
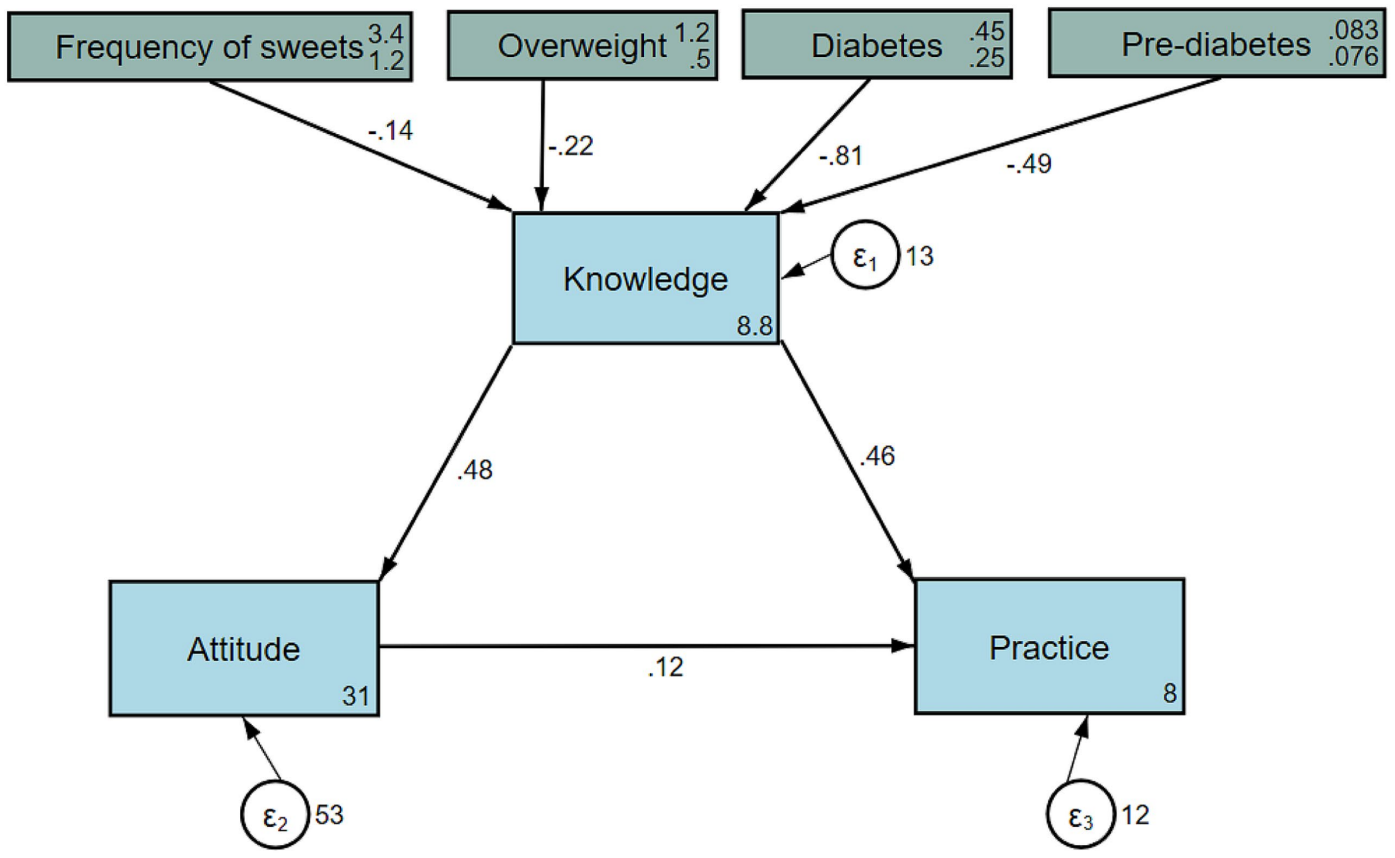
Path analysis showing the associations between demographic characteristics and KAP. All variables are observed variables. The direction of causality is indicated by single-headed arrows. The standardized path coefficients are presented alongside the arrows.

Pearson’s correlation analysis showed that knowledge was positively correlated with attitude (*r* = 0.229, *p* < 0.001) and practice (*r* = 0.467, *p* < 0.001). Additionally, a positive correlation was found between attitude and practice (*r* = 0.312, *p* < 0.001) (Table 2). The path analysis model had a satisfactory model fit (CFI = 0.947, RMSEA = 0.050; TLI = 0.901; SRM*R* = 0.033) (Table 3) Moreover, path analysis showed that diabetes diagnosis was directly associated with lower knowledge (path coefficient = −0.81, 95%CI: −1.58, −0.05; *p* = 0.038) and indirectly associated with lower practice (path coefficient = −0.42, 95%CI: −0.82, −0.02; *p* = 0.041). Knowledge was directly and positively associated with attitude (path coefficient = 0.48, 95%CI: 0.29, 0.66; *p* < 0.001) and practice (path coefficient = 0.46, 95%CI: 0.37, 0.55; *p* < 0.001). Besides, significantly indirect association was observed between knowledge and practice (path coefficient = 0.06, 95%CI: 0.03, 0.08; *p* < 0.001). In addition, the attitude was directly and positively associated with practice (path coefficient = 0.12, 95%CI: 0.07, 0.16; *p* < 0.001) (Figure 3; Table 4).

**Table 2:** Correlation analysis of knowledge, attitude and practice dimensions among participants.

	Knowledge	Attitude	Practice
Knowledge	1		
Attitude	0.229 (*P* < 0.001)	1	
Practice	0.467 (*P* < 0.001)	0.312 (*P* < 0.001)	1

**Table 3:** Goodness of fit of path analysis.

	Value	Indicate
RMSEA	0.050	Acceptable
CFI	0.947	Good fit
TLI	0.901	Good fit
SRMR	0.033	Acceptable

RMSEA, root mean square error of approximation; CFI, comparative fit index; TLI, Tucker-lewis index; SRMR: standardized root mean squared residual.

**Table 4:** The direct and indirect estimates of path analysis.

Model paths	Direct effect		Indirect effect	
	*β* (95% CI)	*P*	*β* (95% CI)	*P*
Frequency of sweets consumption → K	−0.14 (−0.46, 0.18)	0.383	–	–
Overweight or not → K	0.22 (−0.71, 0.27)	0.374	–	–
Having diabetes or not → K	−0.81 (−1.58, −0.05)	0.038	–	–
Having pre-diabetes or not→ K	−0.49 (−1.73, 0.74)	0.432	–	–
K → A	0.48 (0.29, 0.66)	<0.001	–	–
Frequency of sweets consumption → A	–	–	−0.07 (−0.22, 0.09)	0.390
Overweight or not → A	–	–	−0.11 (−0.34, 0.13)	0.381
Having diabetes or not → A	–	–	−0.39 (−0.78, 0.01)	0.055
Having pre-diabetes or not→ A	–	–	−0.24 (−0.83, 0.36)	0.437
K → P	0.46 (0.37, 0.55)	<0.001	0.06 (0.03, 0.08)	<0.001
A → P	0.12 (0.07, 0.16)	<0.001	–	–
Frequency of sweets consumption → P	–	–	−0.07 (−0.24, 0.09)	0.385
Overweight or not → P	–	–	−0.11 (−0.37, 0.14)	0.375
Having diabetes or not → P	–	–	−0.42 (−0.82, −0.02)	0.041
Having pre-diabetes or not→ P	–	–	−0.26 (−0.90, 0.38)	0.433

The variables K, A, and P represent knowledge, attitude, and practice, respectively. The directional arrow signifies the pathway from the arrowhead to the arrowend. For instance, “K → A” denotes the pathway from knowledge to attitude.

### Sensitivity analysis

To avoid the potential impacts of conflicts in the benefits of non-nutritive sweeteners, we removed the question 7 in the knowledge dimension. Participants achieved average knowledge score of 6.22 ± 2.86 (possible range: 0–13). Pearson’s correlation analysis showed that knowledge (*r* = 0.437, *p* < 0.001) and attitude (*r* = 0.312, *p* < 0.001) were positively correlated with practice. Besides, knowledge was marginally correlated with attitude (*r* = 0.084, *p* = 0.073) (Supplementary Table S4). Moreover, path analysis showed that diabetes diagnosis was directly associated with lower knowledge (path coefficient = −0.72, 95%CI: −1.29, −0.11; *p* = 0.046), and indirectly associated with lower attitude (path coefficient = −0.16, 95%CI: −0.39, −0.01; *p* = 0.047) and practice (path coefficient = −0.43, 95%CI: −0.81, −0.07; *p* = 0.046). Besides, knowledge showed directly (path coefficient = 0.57, 95%CI: 0.47, 0.68; *p* = 0.008) and indirectly positive associations (path coefficient = 0.03, 95%CI: 0.01, 0.07; *p* = 0.029) with practice. Moreover, the attitude was directly and positively associated with practice (path coefficient = 0.15, 95%CI: 0.11, 0.19; *p* = 0.007) (Supplementary Table S5).

## Discussion

The present study revealed that the population with reduced sugar intake requirement had poor knowledge, negative attitudes, and suboptimal practices toward non-nutritive sweeteners. Furthermore, significant positive correlations were observed among KAP scores, while a negative association was found between diabetes diagnosis and KAP. Our findings can facilitate the enhanced health management of non-nutritive sweeteners among individuals aiming to reduce sugar intake.

Consistent with our findings, about half of the participants in the United Kingdom expressed a high-risk perception regarding non-nutritive sweeteners and demonstrated limited awareness of relevant regulations (12). Besides, around half of the patients with diabetes demonstrated moderate knowledge and attitude toward non-nutritive sweetener consumption in Iran (13). Therefore, educational programs and evidence-based counseling or support groups are recommended for those defects in KAP scores.

Participants demonstrated sufficient awareness of non-nutritive sweeteners in commonly consumed foods like chewing gum, cakes, and beverages, which is consistent with scientific evidence supporting the wide use of non-nutritive sweeteners as a sugar substitute (20, 21). However, participants might lack a complete understanding of sweetener consumption. Firstly, only 15.54% of participants correctly identified commonly used natural sweeteners in China, highlighting the need for better consumer education and food labeling practices. Clear and accurate labels that provide information about sweetener types can help individuals make informed choices. Secondly, approximately one-third of participants demonstrated awareness of the potential benefits associated with the use of non-nutritive sweeteners, such as weight control, lower risk of chronic diseases, and the inhibition of blood sugar elevation, which is consistent with existing evidence supporting the use of non-nutritive sweeteners in promoting specific health outcomes (22–24). In order to empower individuals to make well-informed choices about their consumption, our findings highlight the importance of enhancing education and communication regarding non-nutritive sweeteners, including their potential advantages and limitations.

The findings from the attitude dimension indicated that participants expressed significant concerns regarding the potential health impact of sweetener consumption. Besides, the varied consensus on the complete safety of non-nutritive sweeteners indicated a lack of confidence in their overall safety, which might stem from inadequate understanding of the regulatory assessments and the debated health implications associated with sweeteners. Addressing these concerns necessitates targeted educational campaigns to elucidate safety regulations of non-nutritive sweeteners and medical evidence based on patient-specific conditions. Furthermore, only a limited proportion of participants agreed on the full sucrose replacement by non-nutritive sweeteners. One plausible explanation was the inconclusiveness of existing scientific evidence on the long-term health effects of non-nutritive sweeteners. Future experimental studies are needed to establish the causality between long-term use of the sweeteners and health implications (8, 25).

Most partcipants preferred food products labeled “zero sugar” or “zero energy.” Besides, 52.3% of respondents were concerned about the functional properties of non-nutritive sweeteners when choosing sweetened foods, thus suggesting that participants considered factors beyond taste, such as reduced calories or specific health claims, when making food choices. However, only a small percentage of participants (17.51%) reported frequently consuming non-nutritive sweeteners to replace sucrose in their diet, highlighting a discrepancy between preferences and actual behavior regarding sweetener consumption. Factors like taste preferences, cultural influences, availability of alternatives, and personal beliefs about the risks or benefits of non-nutritive sweeteners may shape participants’ dietary choices (26). The considerable attention to the potential risks associated with non-nutritive sweeteners reflected a demand for clear and transparent information on health-related concerns. Additionally, participants expressed concerns regarding non-nutritive sweeteners that deliver sweetness without affecting caloric intake or blood glucose levels (27). Moreover, their preference for natural sweeteners like stevia and mogroside reflected the current trend of seeking minimally processed and healthier food options that align with their dietary goals (27, 28).

The correlation analysis and path analysis results demonstrated that higher knowledge about non-nutritive sweeteners may contribute to more positive attitudes and better adherence to recommended practices. These findings aligned with the theory of planned behavior, which suggests that attitudes are influenced by beliefs about the consequences of behavior (29). However, the correlation coefficients ranged from 0.229 to 0.467, indicating weak associations. One plausible explanation could be the multifaceted nature of dietary behaviors. Factors such as taste preferences, cultural influences, socio-economic status, and individual differences in health beliefs may have pivotal roles in shaping KAP scores toward non-nutritive sweeteners. Regular employees had significantly higher knowledge and practice scores than other occupations, which could derive from more exposure to health-related information through employee health programs (30). Besides, higher-income participants have greater access to diverse food options and nutritional education, all of which contribute to informed decisions about sweetener consumption (31). The lower knowledge among individuals with diabetes might be due to the extensive information primarily focused on blood sugar control, medication adherence, and dietary restrictions. Consequently, their exposure to educational materials or interventions targeting non-nutritive sweeteners could be limited. The indirect association between diabetes and lower practice toward non-nutritive sweeteners could be influenced by prioritizing other dietary modifications, such as reducing overall carbohydrate intake or monitoring portion sizes, over using non-nutritive sweeteners. Additionally, concerns about the potential impact of non-nutritive sweeteners on blood sugar control might increase hesitancy among individuals with diabetes to adopt these substitutes. In the sensitivity analysis, participants’ knowledge scores were still poor, and the main results were consistent with prior findings. However, the correlation between knowledge and attitude was changed to not significant. It suggested that knowledge regarding the benefits of non-nutritive sweeteners could impact public attitude toward it. The recent WHO guideline challenges previous assumptions about the benefits of NNS, necessitating a reevaluation of our findings in the context of evolving health recommendations. As scientific understanding advances, ongoing research will be crucial in informing public health strategies and fostering a more nuanced understanding of the role of non-nutritive sweeteners in our diets (32, 33).

There are several limitations in the present study that need to be acknowledged. Firstly, the representativeness of sample selection might restrict the generalizability of findings to a broader population. Secondly, the use of self-developed cross-sectional questionnaires raises concerns about the reliability and validity of the data, as well as the ability to establish causality or temporal relationships. Thirdly, questionnaires collected from face-to-face interviews were electronically recorded, posing a challenge in discerning a clear demarcation between the two strategies employed. Lastly, the QR codes were distributed openly, which prevented the tracking of questionnaire visits and hindered the calculation of the response rate.

In conclusion, the populations with reduced sugar intake requirements showed poor knowledge, negative attitudes, and suboptimal practices toward non-nutritive sweeteners. To optimize the utilization of non-nutritive sweeteners in accordance with medical prescriptions, especially for individuals with diabetes, tailored educational interventions may be designed for participants with lower KAP.

## Data availability statement

The original contributions presented in the study are included in the article/Supplementary material, further inquiries can be directed to the corresponding authors.

## Ethics statement

The studies involving humans were approved by the Medical Ethics Committee of the PLA General Hospital (KY2022-025). The studies were conducted in accordance with the local legislation and institutional requirements. The participants provided their written informed consent to participate in this study.

## Author contributions

QC: Formal analysis, Writing – original draft. YZ: Formal analysis, Writing – original draft. HL: Formal analysis, Writing – review & editing.
