# Supplementary material for: Knowledge, attitude, and practice toward non-nutritive sweeteners among the population with reduced sugar intake requirement
Source: Front Nutr. 2024 Jan 5;10:1268599. doi: 10.3389/fnut.2023.1268599 (PMC10796998; doi:10.3389/fnut.2023.1268599)
Supplement: Supplementary file 1 [file Data_Sheet_1.docx]

**[Supplementary](javascript:;) Table 1**. Distribution of knowledge dimension responses

|  | a. Correct | b. Wrong | c. Unsure |
| --- | --- | --- | --- |
| **1. Sweeteners can be divided into natural and artificial sweeteners according to their source, and also into nutritive and non-nutritive sweeteners according to their nutritional value** | 293 (64.11) | 35 (7.66) | 129 (28.23) |
| **2. Sugar substances such as glucose, fructose, sucrose, maltose, starch sugar and lactose are also natural sweeteners, but they are not usually considered as sugar substitutes** | 227 (49.67) | 91 (19.91) | 139 (30.42) |
| **3. Natural sweeteners commonly used in China include stevia, mogroside and allulose (False)** | 179 (39.17) | 71 (15.54) | 207 (45.3) |
| **4. Artificial sweeteners commonly used in China include xylitol, aspartame, sodium cyclamate, etc.** | 289 (63.24) | 42 (9.19) | 126 (27.57) |
| **5. The sweetness of natural sweeteners is usually similar to that of cane sugar, while the sweetness of artificial sweeteners can be tens to hundreds of times that of cane sugar** | 261 (57.11) | 46 (10.07) | 150 (32.82) |
| **6. Sweeteners do not contain energy and do not affect blood sugar, so they can be eaten freely (False)** | 9 (1.97) | 347 (75.93) | 101 (22.1) |
| **7. The benefits of using sweeteners include** |  |  |  |
| **Weight control** | 151 (33.04) | 184 (40.26) | 122 (26.7) |
| **Reduction of tooth decay** | 173 (37.86) | 178 (38.95) | 106 (23.19) |
| **Reducing the risk of chronic diseases** | 158 (34.57) | 160 (35.01) | 139 (30.42) |
| **Not raising blood sugar** | 162 (35.45) | 177 (38.73) | 118 (25.82) |
| **8. Sweeteners are commonly used in foods such as chewing gum, cake and beverages** | 358 (78.34) | 32 (7) | 67 (14.66) |
| **9. In addition to reducing sugar intake, some natural sweeteners have the function of regulating blood sugar and lipid metabolism.** | 177 (38.73) | 96 (21.01) | 184 (40.26) |
| **10. Potential hazards of excessive intake of sweeteners include** |  |  |  |
| **Carcinogenic** | 183 (40.04) | 86 (18.82) | 188 (41.14) |
| **Addiction** | 252 (55.14) | 75 (16.41) | 130 (28.45) |
| **Diminished energy perception** | 262 (57.33) | 44 (9.63) | 151 (33.04) |
| **Disruption of intestinal flora** | 261 (57.11) | 45 (9.85) | 151 (33.04) |

[**Supplementary**](javascript:;) **Table 2**. Distribution of attitude dimension responses

|  | a. Strongly agree | b. Agree | c. Neutral | d. Disagree | e. Strongly disagree | Mean ± SD |
| --- | --- | --- | --- | --- | --- | --- |
| **1. Sweetened foods do not taste any different from sugary foods** | 32 (7) | 107 (23.41) | 122 (26.7) | 155 (33.92) | 41 (8.97) | 2.86 ± 1.09 |
| **2. There is no difference between the use of artificial sweeteners and natural sweeteners in food** | 27 (5.91) | 84 (18.38) | 108 (23.63) | 159 (34.79) | 79 (17.29) | 2.61 ± 1.14 |
| **3. I am concerned that long-term consumption of sweeteners may cause me to become addicted to them** | 97 (21.23) | 169 (36.98) | 106 (23.19) | 69 (15.1) | 16 (3.5) | 2.43 ± 1.09 |
| **4. I am concerned that consumption of sweeteners may have an impact on my health** | 142 (31.07) | 162 (35.45) | 104 (22.76) | 36 (7.88) | 13 (2.84) | 2.16 ± 1.04 |
| **5. Sweetened foods are more suitable for patients with diabetes and obese/overweight people than sugary foods** | 58 (12.69) | 140 (30.63) | 149 (32.6) | 76 (16.63) | 34 (7.44) | 3.25 ± 1.10 |
| **6. Sweetened foods could provide me with the same level of satisfaction compared to sugary foods** | 72 (15.75) | 163 (35.67) | 145 (31.73) | 61 (13.35) | 16 (3.5) | 3.47 ± 1.02 |
| **7. Eating sweetened foods could reduce the guilt I experienced when eating sweets** | 57 (12.47) | 138 (30.2) | 126 (27.57) | 108 (23.63) | 28 (6.13) | 3.19 ± 1.12 |
| **8. Sweetened foods could help me control and reduce my calorie intake** | 68 (14.88) | 135 (29.54) | 145 (31.73) | 80 (17.51) | 29 (6.35) | 3.29 ± 1.11 |
| **9. Consuming sweetened foods is beneficial to me personally** | 33 (7.22) | 104 (22.76) | 173 (37.86) | 111 (24.29) | 36 (7.88) | 2.97 ± 1.04 |
| **10. I believe that sweeteners are perfectly safe for the health of the consumer** | 16 (3.5) | 42 (9.19) | 159 (34.79) | 160 (35.01) | 80 (17.51) | 2.46 ± 1.00 |
| **11. The benefits of consuming sweetened foods outweigh the risks** | 36 (7.88) | 93 (20.35) | 191 (41.79) | 88 (19.26) | 49 (10.72) | 2.95 ± 1.07 |
| **12. I believe that sweeteners can be completely replaced for sucrose in food products** | 23 (5.03) | 76 (16.63) | 146 (31.95) | 138 (30.2) | 74 (16.19) | 2.64 ± 1.09 |

[**Supplementary**](javascript:;) **Table 3**. Distribution of practice dimension responses

|  | a. Always n (%) | b. Often n (%) | c. Usually n (%) | d. Occassionally n (%) | e. Never n (%) | Mean ± SD |
| --- | --- | --- | --- | --- | --- | --- |
| **1. The consumption frequency of non-nutritive sweeteners as a substitute for sucrose in my daily dietary intake is substantial** | 11 (2.41) | 69 (15.1) | 137 (29.98) | 163 (35.67) | 77 (16.85) | 2.51 ± 1.02 |
| **2. I would choose sweetened foods over sugary foods** | 31 (6.78) | 91 (19.91) | 147 (32.17) | 124 (27.13) | 64 (14) | 2.78 ± 1.12 |
| **3. I will pay attention to the use of sweeteners when buying food.** | 59 (12.91) | 136 (29.76) | 107 (23.41) | 81 (17.72) | 74 (16.19) | 3.05 ± 1.28 |
| **4. When choosing sweetened foods, I would consider the potential health effects it may cause** | 69 (15.1) | 162 (35.45) | 116 (25.38) | 74 (16.19) | 36 (7.88) | 3.34 ± 1.15 |
| **5. I prefer to choose foods that are labelled as "zero sugar" or "zero energy** | 134 (29.32) | 166 (36.32) | 102 (22.32) | 42 (9.19) | 13 (2.84) | 3.80 ± 1.05 |
| **6. When choosing sweetened foods, I am more concerned about the taste and texture of the food** | 64 (14) | 156 (34.14) | 164 (35.89) | 53 (11.6) | 20 (4.38) | 3.12 ± 1.06 |
| **7. When choosing sweetened foods, I am more concerned about the function of the sweetener added** | 61 (13.35) | 178 (38.95) | 147 (32.17) | 53 (11.6) | 18 (3.94) | 3.10 ± 1.20 |

[**Supplementary**](javascript:;) **Table 4**. Correlation analysis of knowledge, attitude and practice dimensions among participants (sensitivity analysis)

|  | Knowledge | Attitude | Practice |
| --- | --- | --- | --- |
| Knowledge | 1 |  |  |
| Attitude | 0.084 (*P*=0.073) | 1 |  |
| Practice | 0.437 (*P*<0.001) | 0.312 (*P*<0.001) | 1 |

[**Supplementary**](javascript:;) **Table 5**. The direct and indirect estimates of path analysis (sensitivity analysis)

| Model paths | Direct Effect |  | Indirect effect |  |
| --- | --- | --- | --- | --- |
|  | β (95% CI) | *P* | β (95% CI) | *P* |
| Frequency of sweets consumption → K | -0.05 (-0.27, 0.23) | 0.630 | - | - |
| Overweight or not → K | -0.05 (-0.82, 0.70) | 0.962 | - | - |
| Having diabetes or not → K | -0.72 (-1.29, -0.11) | 0.046 | - | - |
| Having pre-diabetes or not→ K | 0.36 (-0.15, 0.81) | 0.364 | - | - |
| K → A | 0.22 (0.06, 0.44) | 0.083 | - | - |
| Frequency of sweets consumption → A | - | - | -0.01(-0.07, 0.05) | 0.458 |
| Overweight or not → A | - | - | -0.01(-0.22, 0.19) | 0.825 |
| Having diabetes or not → A | - | - | -0.16(-0.39, -0.01) | 0.047 |
| Having pre-diabetes or not→ A | - | - | 0.08(-0.04, 0.23) | 0.162 |
| K → P | 0.57 (0.47, 0.68) | 0.008 | 0.03(0.01, 0.07) | 0.029 |
| A → P | 0.15 (0.11, 0.19) | 0.007 | - | - |
| Frequency of sweets consumption → P | - | - | -0.03(-0.16, 0.14) | 0.630 |
| Overweight or not → P | - | - | -0.03(-0.53, 0.42) | 0.963 |
| Having diabetes or not → P | - | - | -0.43(-0.81, -0.07) | 0.046 |
| Having pre-diabetes or not→ P | - | - | 0.22(-0.86, 0.52) | 0.351 |
